# Bilateral Lung Transplantation in Kartagener’s Syndrome and Situs Inversus

**DOI:** 10.7759/cureus.35785

**Published:** 2023-03-05

**Authors:** Muhammad Asad Faruqi, Suresh Keshavamurthy, Karl D Hillenbrand, Michael Anstead, Sravanthi Nandavaram

**Affiliations:** 1 Pulmonary and Critical Care Medicine, University of Kentucky College of Medicine, Lexington, USA; 2 Surgery/Cardiothoracic, University of Kentucky, Lexington, Kentucky, USA; 3 Anesthesia and Critical Care, University of Kentucky College of Medicine, Lexington, USA; 4 Pulmonology, University of Kentucky, Lexington, USA; 5 Pulmonary Critical Care, Sleep Medicine and Lung Transplantation, University of Kentucky, Lexington, USA

**Keywords:** veno-arterial-venous extracorporeal membrane oxygenation, bilateral anterolateral thoracotomy, situs inversus, kartagener’s syndrome, primary ciliary dyskinesia

## Abstract

Kartagener’s syndrome (KS) is a genetic disorder and a subgroup of primary ciliary dyskinesia characterized by situs inversus, chronic sinusitis and bronchiectasis. Patients with KS can develop severe bronchiectasis with end-stage lung disease due to recurrent pulmonary infections. Lung transplantation is a treatment option with good outcomes reported in the literature. Lung transplantation in such patients can be technically challenging given the dextrocardia, bronchial asymmetry and anatomical variation of major vascular structures due to situs inversus. We present a case of a 45-year-old male with KS complicated by recurrent infections and chronic respiratory failure, who successfully underwent a bilateral sequential lung transplant (BSLTx). Because of repeated infections and severe bronchiectasis, the patient's quality of life was impaired, and he was oxygen dependent. As a definitive treatment, successful lung transplantation led to a reversal of hypoxic respiratory failure and the patient's symptoms markedly improved, reinforcing data in the literature to consider lung transplantation in this patient population.

## Introduction

Primary ciliary dyskinesia (PCD) is the congenital impairment of muco-ciliary clearance [1]. It is generally inherited as an autosomal recessive disease; however, autosomal dominant and X-linked inheritance has been reported. This inherited disease has an equal prevalence in males and females [2]. Over 50 genetic mutations associated with PCD have been reported which affect different parts of the ciliary structures or function [3]. About 50% of patients with PCD will have situs inversus which is characterized by a complete reversal of viscera and the circulatory system. The majority of patients with PCD will have a presentation in childhood (median age of diagnosis 5 to 5.5 years), but some can present in adulthood (median age of diagnosis 22 years). There is no specific treatment for Kartagener’s syndrome (KS) and management is tailored according to every patient's disease process. It includes preventing and treating recurrent pneumonia, which eventually leads to bronchiectasis. Airway clearance and targeted therapy with antibiotics are the mainstays of the management of these patients, in addition to vaccinations [4]. Patients suffering from ciliary dysmotility most frequently present with recurrent upper and lower respiratory tract infections [5]. KS is a subgroup of PCD, characterized by the triad of situs inversus, chronic sinusitis, and bronchiectasis, and has a variable prevalence of about one in 10,000 to 40,000 individuals around the world [6,7]. Diagnosis involves a high index of suspicion in patients who may have recurrent infections of the respiratory tract, otitis, infertility, etc. There is no gold standard test for diagnostic evaluation. Compatible history along with some other tests like nasal nitric oxide, high-speed video-microscopy analysis, electron microscopy, and genetic testing are employed to confirm the diagnosis [8]. KS can lead to chronic respiratory insufficiency with end-stage lung disease and lung transplantation (LT) is a feasible treatment option in this subset of patients. Though there have been reports of LT in patients with KS, there are no specific guidelines regarding technical modifications required for the procedure in this patient population. The first ever heart and lung transplant was performed on a KS patient back in 1992 and since then, many advances have been made in this field [9]. LT for KS is rare. A review of the United Network for Organ Sharing registry showed that 12 bilateral lung transplants have been performed between 1987 and 2015. Some cases reported substantial intraoperative modifications, while others reported that no adjustments were necessary [10,11]. Our case report aims to highlight this treatment option which exists for patients who have chronic severe respiratory insufficiency and increased morbidity. A bilateral lung transplant can greatly improve this population's quality of life.

## Case presentation

**Figure 1:**
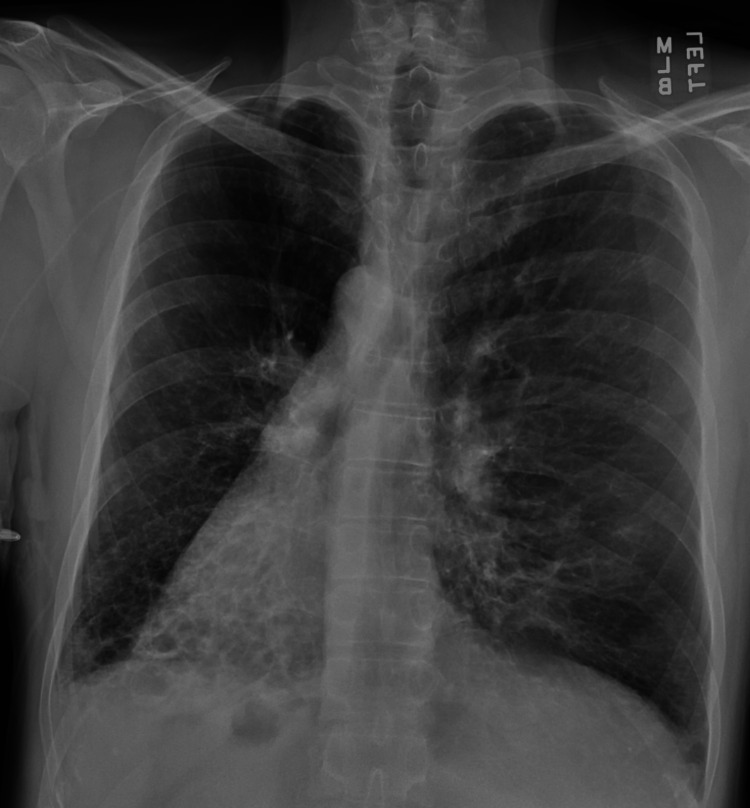
Chest x-ray showing dextrocardia

**Figure 2:**
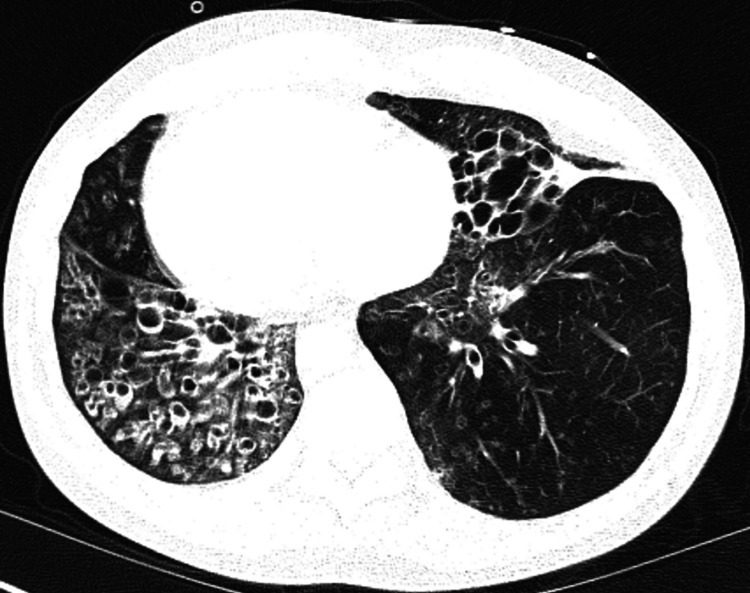
CT scan of the chest demonstrating severe bronchiectasis.

**Figure 3:**
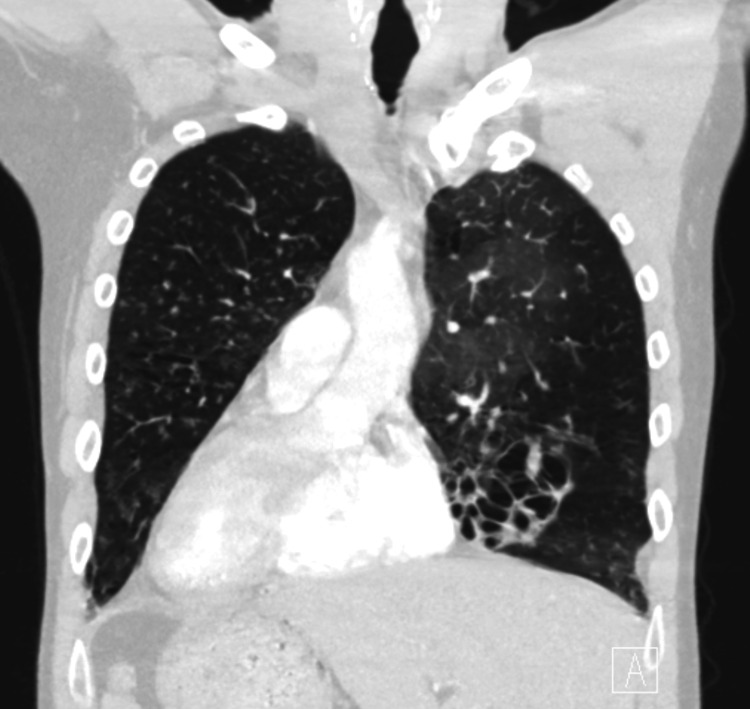
CT scan showing dextrocardia and bronchiectasis

A 45-year-old Caucasian male with no pertinent past medical history with KS, whose clinical course was complicated with recurrent pulmonary infections, on long-term oxygen therapy up to 5 liters, was referred for LT. The patient was diagnosed in early adulthood and had been battling with recurrent pneumonia requiring frequent intravenous antibiotics, chest physiotherapy and hospital admissions. He did not have any smoking history or any other pertinent exposures that could cause lung disease. The patient lived on a farm and took care of cattle. He had a pet dog. The patient did not have any cardiac issues. The patient was seen by transplant pulmonology. On evaluation, the patient’s pulmonary function tests revealed severe obstruction and reduced diffusion capacity. Transthoracic echocardiography showed normal left ventricular function but a mildly dilated right atrium and mildly reduced right ventricular function. Right heart catheterization (RHC) revealed secondary pulmonary hypertension (pulmonary artery pressure 54/20 mmHg, mean 34 mmHg). Imaging including chest x-rays and computerized tomography (CT) scans showed dextrocardia and severe bronchiectasis (Figures 1-3). Due to the patient's worsening respiratory failure and frequent pneumonia, it was decided to list him for LT and eventually, a donor became available. As described in the introduction, LT being a definitive treatment for end-stage KS, the patient underwent bilateral sequential LT.

**Figure 4:**
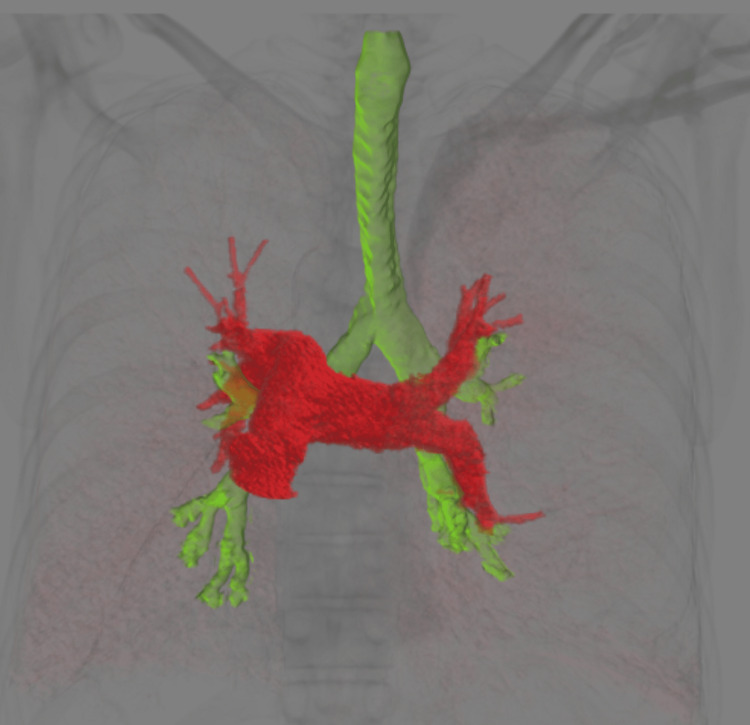
3D reconstruction of airways and vessels shows hyp-arterial bronchus on the right side due to situs inversus.

**Figure 5:**
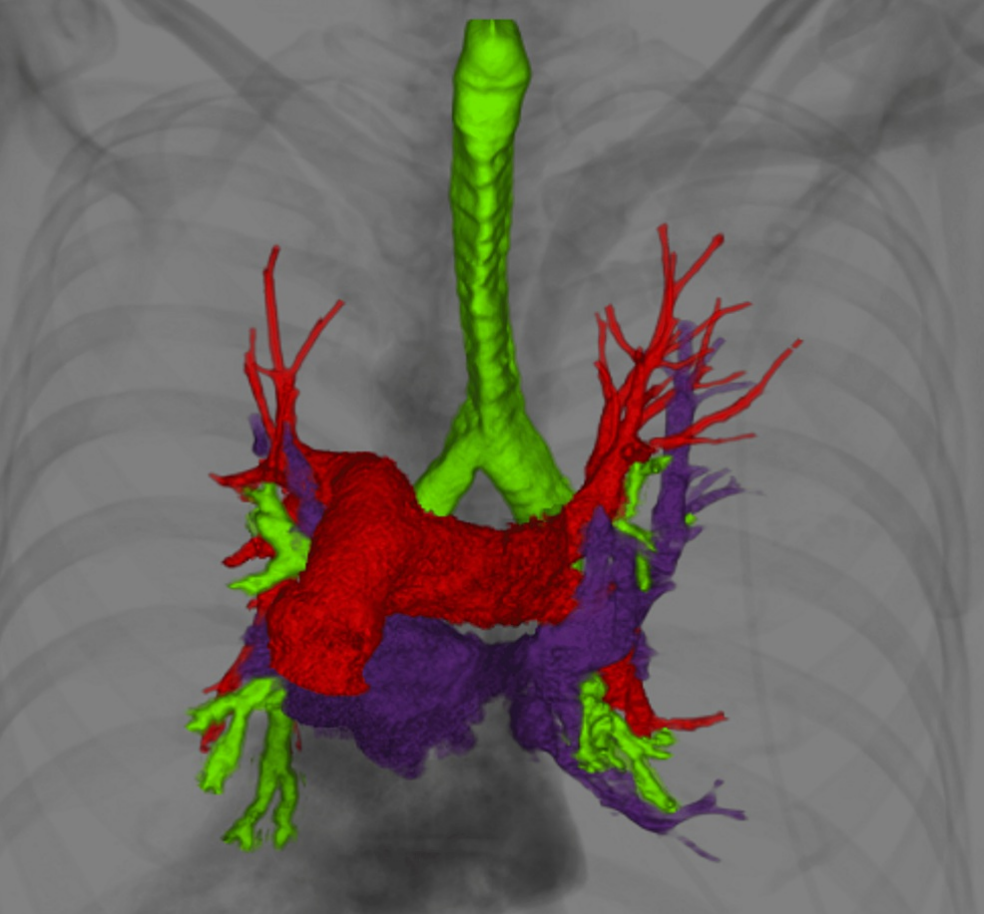
3D reconstruction of the airways and vessels shows left-sided shorter ep-arterial bronchus.

Donor-recipient size matching was done to avoid right lung volume reduction. 3D reconstruction was done to evaluate the vascular anatomy in relation to the bronchi. It showed right-sided hyp-arterial bronchus and left sided shorter ep-arterial bronchus (Figures 4, 5).

The patient was taken to the OR and a left-sided double lumen endotracheal tube was placed in the anatomical right mainstem bronchus because of reverse anatomy in the recipient. Given the moderately elevated pulmonary artery pressures, the patient was electively placed on central veno-arterial extra corporeal membrane oxygenation (ECMO) with aortic and morphological right atrial appendage cannulation. After initiation of ECMO, right native lung was deflated. The donor's right lung was implanted in a standard fashion. No size mismatch was encountered. On the left side bronchial anastomosis was performed in standard fashion and donor pericardium was tacked over the bronchial anastomosis. The pulmonary arterial anastomosis required extensive mobilization of the donor and recipient pulmonary arteries and the donor PA had to be divided in beveled fashion to accommodate for the altered anatomy.

**Figure 6:**
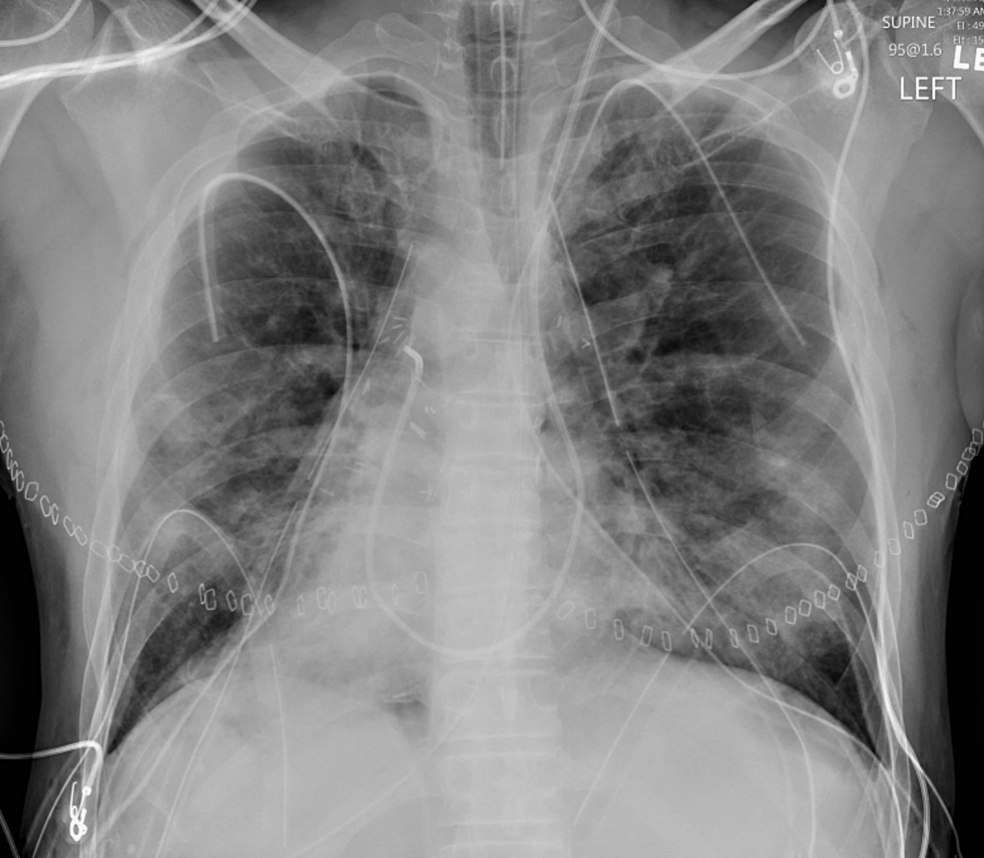
Chest x-ray post operatively shows newly implanted lungs.

**Figure 7:**
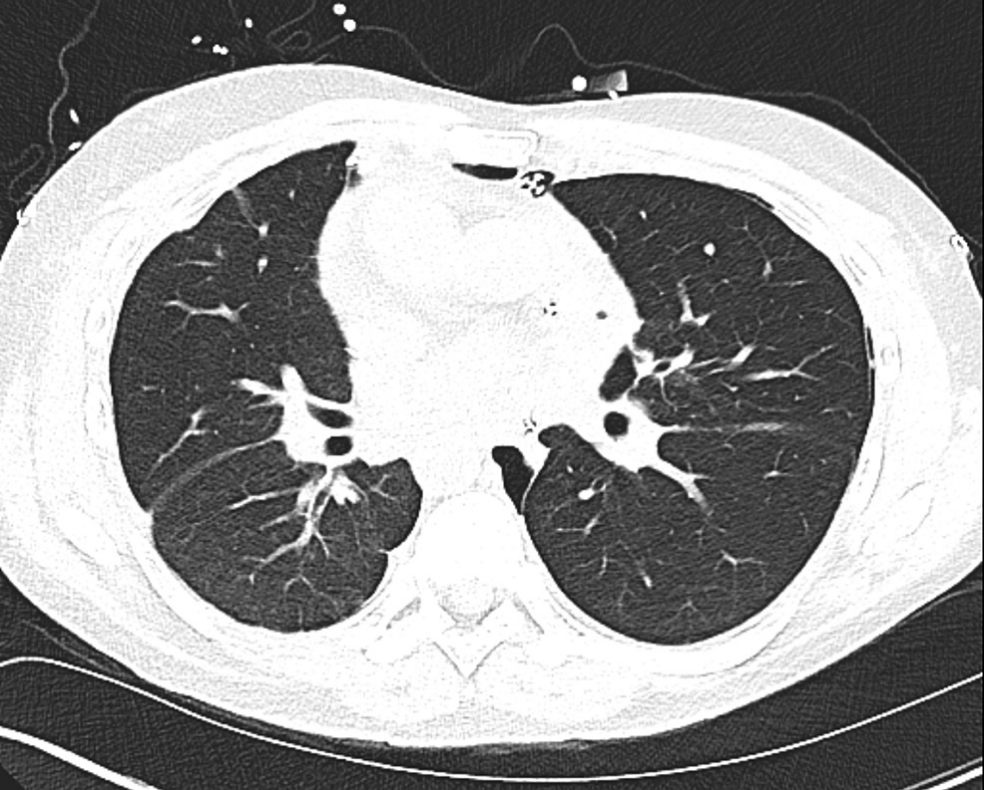
CT chest prior to hospital discharge

**Figure 8:**
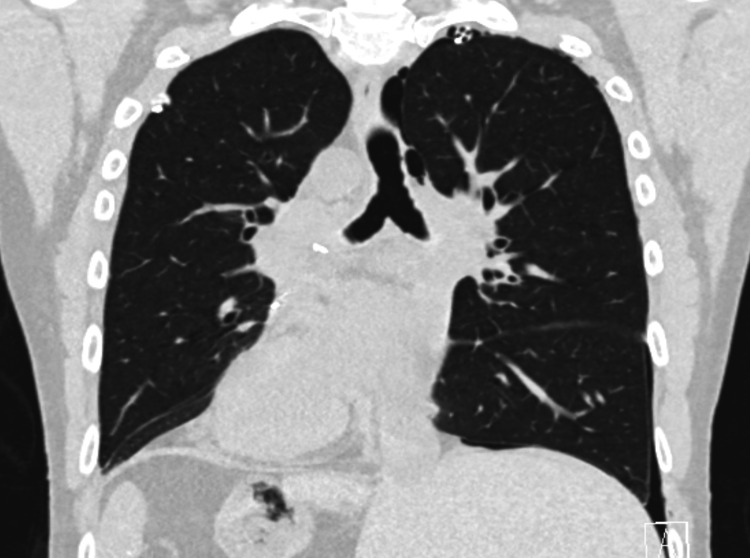
CT chest demonstrating transplanted lungs in the patient with Kartagener's syndrome

**Figure 9:**
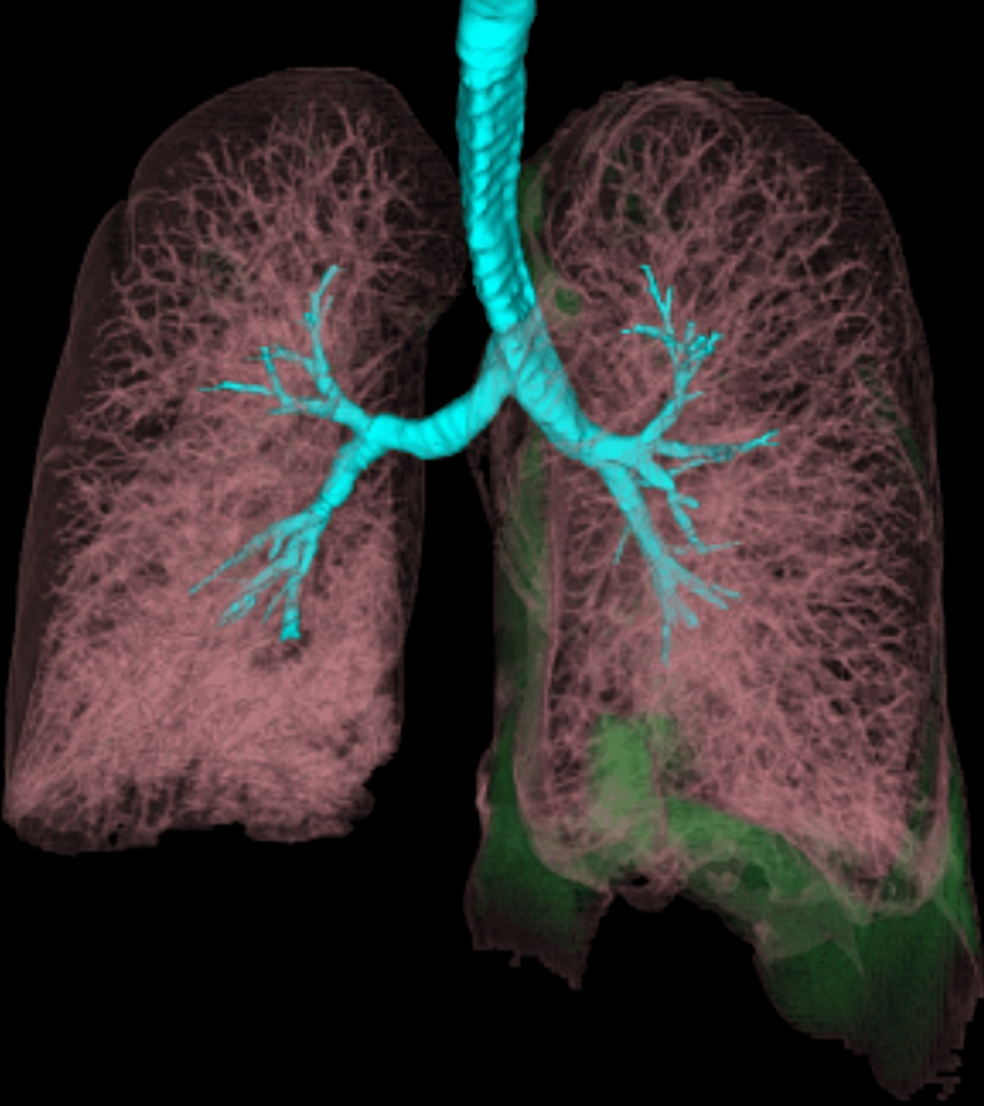
3D reconstruction shows newly transplanted lungs.

Post lung implantation and reperfusion, the patient was weaned off central VA ECMO. Upon chest closure, double lumen tube was exchanged for a single lumen endotracheal tube and bronchoscopy was performed, demonstrating patent bronchial anastomoses. Post operative imaging showed no immediate complications (Figure 6). CT chest and 3D reconstruction image showed normal transplanted lungs (Figures 7-9).

**Figure 10:**
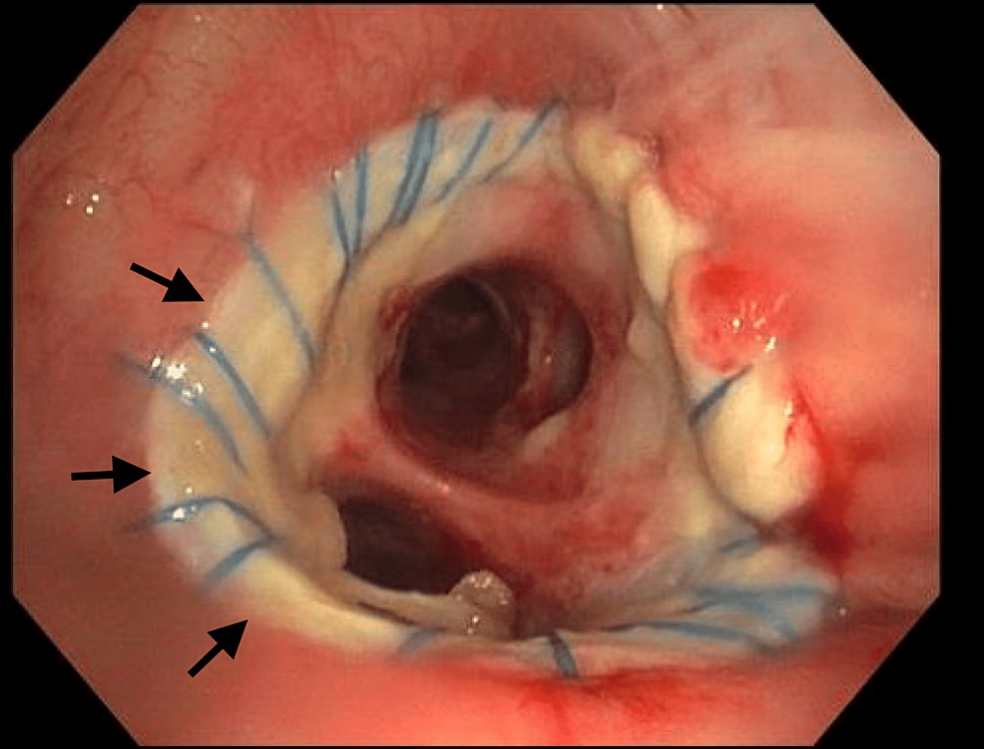
Right mainstem bronchus shown. Arrows point to the anastomosis between donor and recipient bronchi.

**Figure 11:**
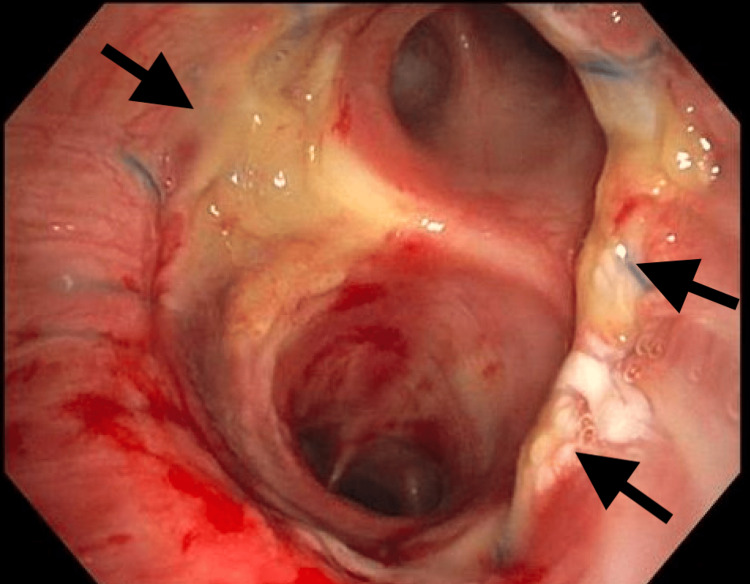
Left mainstem bronchus shown. Arrows point to the anastomosis between donor and recipient bronchi.

The patient was extubated 48 hours post LT and got discharged home on room air within two weeks. Bronchoscopy done post operatively showed patent anastomosis (Figures 10, 11). Surveillance biopsies did not show any acute cellular rejection.

## Discussion

There is a paucity of literature and guidelines regarding LT in patients with KS. The very first heart and lung transplant for a KS patient was documented in 1992 [9]. Double lung transplant (DLTx) is a viable treatment option for end-stage lung disease with KS and has been reported to be successful with reasonable outcomes. Using guidance from this ever-increasing literature, we decided to do a bilateral lung transplant on our patient as well. Situs inversus in KS can pose technical challenges during DLTx. These include a longer anatomical right mainstem bronchus and shorter left mainstem bronchus in the recipient, positioning of the donor and recipient left pulmonary artery, and dextrocardia causing decreased space for the donor right lung. We did not encounter any size mismatch issues and the donor's right lung was easily transplanted into the recipient without requiring donor lobectomy or wedge resection. The left pulmonary arteries of both donor and recipient lungs were mobilized and anastomosed in a beveled fashion which has been described in the literature [12]. In addition, different surgical procedures are described in the literature including “clamshell incision” and bilateral anterolateral thoracotomy without sternal splitting [13]. We used bilateral anterolateral thoracotomy in our patient with success. Utilization of 3D reconstruction images of the lung, bronchial, and pulmonary vascular anatomy along with multi-disciplinary collaborative planning between cardiothoracic surgery, anesthesia, and radiology preoperatively and acceptance of a near similar donor height and lung volumes, ensured a smooth intraoperative course and postoperative course without any complications.

## Conclusions

DLTx in patients with end-stage KS can be performed safely and pre-operative 3D reconstruction images can provide a better insight into the anatomical differences and potential challenges that can be encountered intraoperatively, in the setting of situs inversus and dextrocardia. Bilateral sequential lung transplant (BSLTx) with anterolateral thoracotomy offers a safe approach that forgoes sternal division and associated potential complications.
